# Giardia lamblia infections in children in Ghana

**DOI:** 10.11604/pamj.2016.24.217.8012

**Published:** 2016-07-12

**Authors:** Isaac Anim-Baidoo, Charles Akugbey Narh, Dora Oddei, Charles Addoquaye Brown, Christabel Enweronu-Laryea, Betty Bandoh, Eric Sampane-Donkor, George Armah, Andrew Anthony Adjei, David Nana Adjei, Patrick Ferdinand Ayeh-Kumi, Ben Adu Gyan

**Affiliations:** 1 Department of Medical Laboratory Sciences, School of Biomedical and Allied Health Sciences, University of Ghana, Korle-Bu Campus, Accra, Ghana; 2 Noguchi Memorial Institute for Medical Research, University of Ghana, Legon, Accra, Ghana; 3 Department of Child Health, School of Medicine and Dentistry, University of Ghana, Korle-Bu Campus, Accra, Ghana; 4 Department of Medical Microbiology, School of Biomedical and Allied Health Sciences, University of Ghana, Korle-Bu Campus, Accra, Ghana; 5 Department of Pathology, School of Biomedical and Allied Health Sciences, University of Ghana, Korle-Bu Campus, Accra, Ghana

**Keywords:** Giardia lamblia, assemblage B, Ghana, diarrhoea

## Abstract

**Introduction:**

Though giardiasis is an important public health problem in Ghana, several aspects of its epidemiology, particularly the molecular epidemiology has not been investigated adequately. This could be a major hindrance to effective surveillance and control of giardiasis in the country. The study was carried out to determine the prevalence, risk factors and genotypes of *Giardia lamblia* infecting children at a paediatric hospital in Ghana.

**Methods:**

A total of 485 patients including 365 diarrhoea and 120 non-diarrhoea children were enrolled into the study. Stool samples were collected and analysed for parasite presence using microscopy, ELISA and PCR. Positive samples were subsequently characterized into assemblages by PCR-RFLP, and further confirmed with sequencing of the *glutamate dehydrogenase* (gdh) gene. Epidemiological data on demographic, clinical and behavioral features of the study subjects were also collected.

**Results:**

Prevalence of *G. lamblia* infections in diarrhoea and non-diarrhoea children were 5.8% and 5% respectively (P>0.5). Sequence data confirmed *Giardia lamblia* assemblage B as the predominant genotype in both diarrhoea and non-diarrhoea cases. There was no significant association of G. *lamblia* infection with any of the epidemiological variables investigated.

**Conclusion:**

Our findings suggest that assemblage B could be the predominant genotype causing giardiasis in children. Increased public health education focusing on good sanitary practices, particularly among mothers and children, could decrease the risk of G. *lamblia* infection.

## Introduction

*Giardia lamblia* is an enteric parasite that causes giardiasis, one of the frequent causes of diarrhoea, in humans, pets and livestock [1]. The parasite is distributed globally, and children are more at risk of infection than adults [2]. Several studies have associated these infections with socio-demographic, hygiene, nutritional and immune status of the host and strain of the parasite [1–3]. Giardiasis infections have also been associated with drinking of contaminated tap water, fresh water and the movement of individuals from a non-endemic region to an endemic region [4, 5]. Antenatal studies in Auckland, New Zealand have shown that changing napkins of children was associated with a risk of giardia infection [6]. In developing countries, common risk factors associated with *G. lamblia* infection are socio-demography: improper sanitation, bad personal hygiene, eating of unwashed fruits and vegetables, and drinking of contaminated tap water [2, 7]. Mukherjee et al., [8] showed that in Kolkata, India, there was a relationship between giardiasis and the socio-economic background of the study population. Majority of the diarrhoea patients were of lower socio-economic status and lived in slums, suggesting that infection could be water and/or food borne. Further, children ≤ 5 years of age were most at risk of parasitic infections. A cohort study in rural Egypt suggested that more females were infected than male infants [9]. In Ghana, there have been reported prevalence rates of 9% of Giardiasis among diarrhoea children in the northern sector [10] and 9.7% in the middle belt [11]. A recent hospital-based study conducted in the capital, Accra, recorded a prevalence of 10.1% [12]. The disease seems to be more prevalent among children and thus, the importance of determining possible risk factors among this cohort cannot be overemphasized. Fast expansion of new settlements without adequate potable water supply, especially in urban communities, leave large populations to consume untreated water. This has consequently increased the risk of water-borne infections, including giardiasis in the country. Although no major outbreak of the disease has yet been reported in Ghana, the increasingly high incidence, 24-32%, reported in some hospitals and polyclinics [13] as well as 5.1-46.5%, in some community day-care centers [14, 15] may suggest wide environmental contamination.

*Giardia lamblia* (syn: *G. intestinalis or G. duodenalis*) has 6 distinct genotypes; assemblages A and B are found in humans and other mammals, C/D in dogs, E in livestock, F in cats, and G in rats [16, 17]. Genotyping of Giardia is seminal to determine sources of infection during outbreaks and also could help elucidate possible transmission routes. Restriction Fragment Length Polymorphism (RFLP) has been used as a useful typing tool in epidemiological studies to investigate possible association of identified genotypes of *G. lamblia* with severity of diarrhoea and other clinical symptoms. Giardia assemblage A has been associated with asymptomatic diarrhoea [18] and B as the predominant genotype in diarrhoea cases [19–21]. There is poor epidemiological data on the genotypes of *G. lamblia* in Ghana, probably because studies investigating diarrhoea in the country have focused on enteric bacteria and other protozoans [22]. Though *Cryptosporidium spp*. is an important cause of diarrhoea in Ghana [22], several studies have reported high prevalence of Giardiasis in the country [11, 12]. Using information from these studies, we reasoned that certain risk factors may predispose children, particularly those below 5 years to giardiasis, as reported in other countries. Thus, in the present study, we attempted to define certain risk factors associated with giardiasis in children < 5 years using data gather from a structured questionnaire. This border on education and socio-cultural practices of mothers, food and water sources, breast-feeding practices of mothers, pet ownership, age and sex of child. Using Restriction Fragment Length Polymorphism (RLFP), we genotyped both PCR and ELISA positive samples and confirmed the genotypes with sequencing. We present data, discussing risk factors of *G. lamblia* infection and suggest assemblage B as the predominant genotype causing diarrhoea in Accra, Ghana.

## Methods

### Study site and subjects

This was a hospital-based prospective cross-sectional study conducted between the periods of March, 2010 and June, 2013. The study was conducted at the Princess Marie Louise Children’s hospital (PML) in Accra, Ghana. The hospital is a major paediatric health facility located within the metropolitan area of the city and accessed by people from all parts of Accra [22]. These include people from different socio-cultural, economic and educational background. At the time of undertaking this study, the daily average attendance of patients to the hospital was 143. These included 85 new attendants and 58 old attendants. Hospital records estimated that about 90% of these patients were children below 5 years, of which about 11% reported with diarrhoea and other related problems (PML records). The study population were children ≤ 5 years, who had been hospitalised at PML due to acute diarrhoea and dehydration. Diarrhoea was defined as passage of loose or watery stools in the previous 24 hours and still present at the time of collection of faecal specimen. Patients who developed diarrhoea, after admission and as a result of food intolerance, were excluded from the study. Some children who were admitted in the same hospital, within the period of this study, for medical conditions other than diarrhoea, were included as controls. In all, 485 patients were included in this study; 365 patients with diarrhoea and 120 patients without diarrhoea (controls). Ethical approval (SAHS-Et/SAHS/PSM-ML/1/26A/2010-2011) was obtained from the Research and Ethical Review Committee of the School of Allied Health Sciences, College of Health Sciences, University of Ghana. The inclusion of participants in the study was strictly voluntary and based on informed consent of their guardians or parents (verbal consent), after explaining to them the objectives of the study.

### Collection of socio-demographic and clinical data

Socio-demographic and clinical data were obtained by a study nurse at the hospital ward, using a structured questionnaire. Socio-demographic data included study participants’ residence, sex, age, parent/guardian occupation and educational background, source of drinking water, breastfeeding habits, whether food was prepared at home or obtained from the street, and presence of domestic animals at home. Clinical data included vomiting, malaise, fever, abdominal pain, frequency of passage and consistency of stools, drugs used for treatment or management, as well as duration, if admitted in a hospital.

### Sample collection

A single stool sample was collected from each patient, by his or her parents or guardian, and placed into a clean disposable plastic container with tight fittings. The consistency of the stool was directly observed, classified and recorded by the study nurse as loose, semi-formed, formed, mucoid, slimy, or watery. Each sample was then divided into two portions, with one portion preserved in 10% formalin and the other stored at – 20^°^C.

### Stool microscopy and enzyme immunoassay test

Stool samples preserved in 10% formalin were processed for microscopy by the direct smear method after formal-ether concentration procedures, as previously described [23]. Slides were observed under the microscopy for *G. lamblia* cysts. The enzyme immunoassay test was performed on the -20^°^C stored faecal samples using the Wampole™ *Giardia/Cryptosporidium* Check^®^ ELISA kit (TechLab Inc.) following the manufacturer’s instructions. Results were read both visually by assessing the colour formed in each well of a microtitre plate and quantified by measuring the absorbance at 450nm on a microplate ELISA reader (Labsystems Multiskan MS). The recommended values (cut-off points) were < 0.150 OD_450_, for the negative control, and ≥ 0.500 for the positive control. In the test wells, any value ≥ 0.150 was considered positive, and recorded.

### DNA extraction, RFLP-PCR and sequencing of samples

Giardia DNA was directly extracted and purified from the stool samples using the MO BIO UltraClean^®^ Fecal DNA Isolation kit, following the manufacturer’s instructions. The purified DNA was stored at -40^°^C until further use. *Giardia lamblia glutamate dehydrogenase* (gdh) gene was amplified by a semi-nested PCR following previously described methods [24, 25]. Amplifications were carried out in an Applied Biosystem thermal cycler 2720. Restriction digests were carried out directly on the second-round PCR products in a final volume of 20µL containing 10µL of PCR product, 4U NlaIV (BioLabs), 1X reaction buffer and 0.2µg/µl BSA. Reaction was incubated at 37^°^C for 3 hours. All reactions were controlled with negative and positive samples. PCR and RFLP products were run, alongside 100bp ladder, on 2% agarose gel stained with ethidium bromide. Purification and sequencing of PCR products were performed by Macrogen Inc, Amsterdam, Netherlands. BLAST searches and multi sequence alignments were performed in MEGA V5 [26].

### Statistical analysis

All data were entered into Microsoft Excel 2010 and analyzed using SPSS V17. Descriptive analyses including computation of arithmetic means, frequencies and percentages were done on the study variables. Univariate analysis was performed using independent-sample t-test, Pearson X^2^ test or Fisher’s exact tests, as appropriate. A P-value of < 0.05 was considered statistically significant.

## Results

### Prevalence and risk factors of giardiasis

Among children with diarrhoea, 21/365 (5.8%) were positive for *G. lamblia*. We detected *G. lamblia* in 6/120 (5.0%) of non-diarrhoea children. There was no significant difference in infections between children with diarrhoea and those without it (P>0.5). Table 1 reports on the outcome of analysis of risk factors in relation to *G. lamblia* infection among the study children. Among the diarrhoea children, 7.1% (15/211) males and 3.9% (6/154) females were infected with *G. lamblia* but the difference was not statistically significant (P=0.193). The risk of infection, although not significant (P=0.729), generally, increased with age but was highest (11.1%, 1/9) in children between the ages of 2 and 3 years [OR=3.456]. Infection was generally low for children ≤ 1 year (<5%). For children of mothers/guardians who had domestic animals (pets) at home, 5.8% (9/156) were infected with *G. lamblia*, whilst 5.7% (12/209) of those without pets, were also infected by the parasite. However, the difference between the two groups was not statistically significant (P=0.999). Presence or absence of domestic animals did not pose a risk for infection (OR=1.000). Commonly reared animals included dogs, cats, sheep, goats and chicken. All domestic animals were kept in the houses and thus, we presumed participants might have had frequent contact with them. Out of the 365 diarrhoea cases, we confirmed *G. lamblia* infection in 13/189 (6.9%) in the dry season (December-March), and 8/176 (4.5%) during the rainy season (April-November). We did not see any statistical difference between the two seasons but risk of infection may be influenced by dry or rainy season (P=0.33, OR=1.551, 95% CI=0.627-3.037). Risk of infection appeared to decrease with increasing level of education of the mother/guardian although there was no significant difference (P=0.732) in infection among participants with different qualifications. Children whose mothers or guardians had no formal education were more at risk of infection (OR=2.548). Exclusive breast-feeding, from birth until 6 months old, is widely practised in Ghana. Among diarrhoea cases, we detected *G. lamblia* in stools of children, 5.5% (12/218), whose mothers practised exclusive breast-feeding. We did not observe any statistical difference (P=0.069) between the two groups, exclusive and non-exclusive breast-feeding. Interestingly, children on exclusive breast-feeding appeared to be at risk of infection (OR=2.827). The sources of drinking water for children were in three categories, children who depended solely on Sachet (bagged) water, pipe borne water, and those who depended on both sources. Over 92% of mothers/guardians gave their children sachet water. Among the latter, 5.9% (20/337) had *G. lamblia* in their stools. Only 1 (3.8%) child was infected with *G. lamblia* among the 26 children who depended on pipe-borne water. Infection was independent (P=0.853) of the source of water, but children depending on sachet water were more at risk of infection (OR=1.645). Although infection was not dependent (P=0.469) on the food source, children who ate food cooked at home were less likely to be infected (OR=0.905) as compared to those who depended on street food (OR=1.853).

**Table 1:** Odds ratios and 95% confidence intervals for *G. lamblia* infection in children and associated risk factors [N = 365]

Character/Risk factor		N	N infected	% infected	X^2^	P-value	OR	95% CI
**1. Gender**	Male	211	15	7.1	1.693	0.193	1.888	0.715-4.982
	Female	154	6	3.9				
**2. Age [months]**	< 6	74	3	4.1	2.038	0.729	1.893	0.675-1.956
	6 - 12	165	8	4.8			1.914	0.564-2.432
	13 - 24	107	8	7.5			2.824	0.432-2.854
	25 - 36	9	1	11.1			3.456	0.325-3.654
	37 - 48	10	1	10.2			2.377	0.435-2.547
**3. Education level of mother**	Tertiary	28	2	7.1	1.264	0.732	0.733	0.234-1.035
	Secondary	250	16	6.4			0.914	0.543-1.543
	Primary	40	1	2.5			1.232	0.876-1.546
	None	47	2	4.3			2.548	0.453-2.748
**4. Source of drinking water**	Sachet	337	20	5.9	0.317	0.853	1.645	0.543-1.246
	Pipe-borne	26	1	3.8			0.986	0.234-1.429
	Both	2	0	0			1.000	0.982-1.023
**5. Breast-feeding habits**	Exclusive	218	12	5.5	5.342	0.069	3.148	0.983-3.276
	Not exclusive	131	6	4.6			2.827	0.782-2.901
	N/R	16	3	18.8			1.529	0.916-1.876
**6. Source of food**	Home	217	13	6	2.535	0.469	0.905	0.675-1.456
	Street	10	1	10			2.886	0.564-2.976
	Both	110	4	3.6			1.853	0.692-2.012
	N/R	28	3	10.7			0.543	0.342-1.398
**7. Presence of domestic animals at home**	Yes	156	9	5.8	0.001	0.999	1.000	0.413-2.448
	No	209	12	5.7				
**8. Seasonal variations**	Dry	189	13	6.9	0.915	0.33	1.551	0.627-3.037
	Rainy	176	8	4.5				

Age of *G. lamblia* positive controls: 12-24 months =1; 36-42 months=2; and 42-48 months =3

### Detection of *G. lamblia* in stool samples by PCR and ELISA

**Figure 1:**
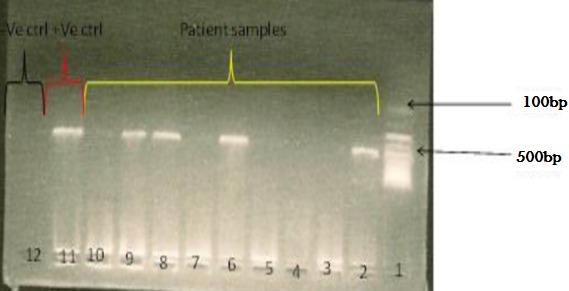
PCR amplification of 490bp fragment of the GDH gene of Giardia lamblia. Lane 1=100bp ladder, lane 2- 10= samples, lane 11= positive control and lane 12= negative control

Of the 365 diarrhoea samples tested, 21 and 4 were positive for ELISA and microscopy, respectively (Table 2). Interestingly, of the 120 non-diarrhoea samples tested, 1 and 6 samples were positive by microscopy and ELISA, respectively. In both cases, all ELISA positive samples also tested positive by PCR, which amplified a 461bp fragment of the gdh gene (Figure 1). In total, 32 samples were positive for *G. lamblia*.

**Table 2:** Detection of *G. lamblia* infection in diarrhoeic and non-diarrhoeic stool by Microscopy and Enzyme immunoassay

*Diarrhoea stool*					
	No. tested	No. Positive	%	No. Negative	%
Microscopy	365	4	1.09	361	98.90
ELISA	365	21	5.75	344	94.25
*Non-diarrhoea stool*					
	No. tested	No. Positive	%	No. Negative	%
Microscopy ELISA	120	1	0.83	119	99.16
	120	6	5.00	114	95

32 samples, non-diarrhoea [7] and diarrhoea [25] were positive for PCR. Two representative sequences showed >95% identity to *G. intestinalis* B

### Sequencing of *G. lamblia*

**Figure 2:**
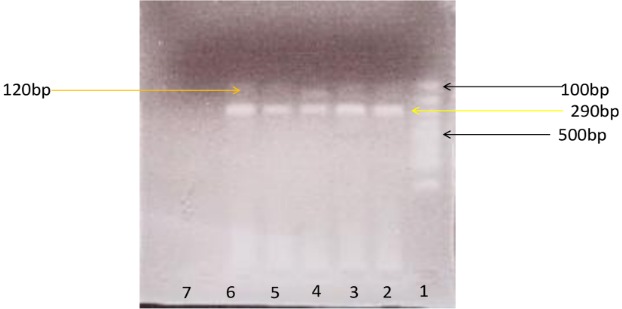
RFLP of Giardia lamblia after digestion with NIaIV. Lane 1=100-bp ladder, lanes 2- 6 = samples and lane 7= negative control. Two products, 150 and 290bp were obtained for all samples

**Figure 3:**
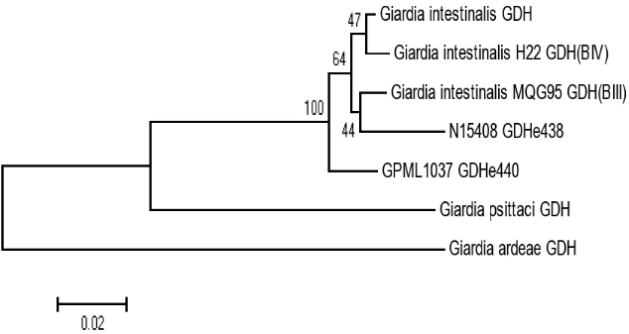
Phylogenetic clustering of Giardia lamblia isolates with reference orthologs

PCR amplification was successful for all 32 ELISA-positive samples. The RFLP with NlaIV enzyme gave a single pattern, 150 and 290bp fragments for all PCR products (Figure 2). This profile was previously reported as genotype B (BIII or BIV) [25]. Although sequencing was attempted for 5 representative PCR products, only two samples, GPML1037 GDHe440 and N15408 GDHe348, had good sequence reads. BLAST searches of the NCBI database confirmed these two sequences, with >95% sequence identity, as *G. lamblia (G. intestinalis)*genotype B. In a separate phylogenetic analysis (Figure 3), sample N15408 GDHe348 formed a clade with MQG95 GDH (BIII), a homolog with GenBank Accession Number JQ700429.

## Discussion

*Giardia lamblia* is a medically important gastrointestinal protozoa associated with diarrhoea, especially in communities without proper sanitation and potable water. Acute diarrhoea is a major cause of mortality and morbidity among children, particularly those below 5 years, in developing countries [27]. In Ghana, previous studies have shown a steady rise in giardiasis with 9%, 9.7% and 10.1% recorded prevalence in 2007, 2011 and 2013, respectively [10–12]. In this study, we recorded a prevalence of 5.8%. This reduction is encouraging and may suggest improved health conditions. Over the past 3 years, Accra has seen a major improvement in sanitation; there have been several public health educational campaigns on personal hygienic practices in the media and in schools. Recently, the government introduced the National Sanitation Day, which organises the populace to clean their immediate surroundings, once every month. A major worry though, is the recorded 5% prevalence in non-diarrhoea cases we observed in this study. Haque et al. [28] made a similar observation, recording a higher prevalence in controls than in diarrhoea cases. Without periodical diagnosis, these people may serve as reservoirs of *G. lamblia* and pass on the parasite to susceptible host. Microscopy for giardia cyst has been the mainstay of diagnosis in health facilities and some research laboratories in Ghana [11]. Using ELISA and PCR, we seem to have relatively improved the detection limit or positivity of *G. lamblia* in stool samples, particularly in non-diarrhoea cases. These data suggest that PCR and/or ELISA may offer superior diagnostic potential to detecting the parasite, particularly in asymptomatic cases. Attempts are on going to further optimize both DNA extraction and PCR protocols for *G. lamblia* detection, in the hope of reducing sample processing and run times. This could be of use in healthcare facilities for prompt and accurate diagnosis. Genotypes A and B have been shown to cause symptomatic infections and this has been extensively reviewed by Dib et al. [29]. Here, all the 32 PCR-positive samples had identical RFLP profile, reported as genotype B [25], which was further confirmed with sequencing. This genotype is predominant in cases of giardiasis and has been associated with related symptoms, including diarrhoea [19–21]. However, among the non-diarrhoea cases (120), we detected *G. lamblia* in 6. This may suggest that infection with Genotype B may not always lead to clinical symptoms. Prior exposure has been shown to confer some degree of protection to subsequent infections [30, 31], and such cases may be reservoirs sustaining transmission to susceptible host, particularly at day-care centers, where children have frequent contact with each other [15, 32, 33]. Interestingly, our data is in contrast to a study by Opintan et al. [22] who identified *Cryptosporidium* as the commonest cause of diarrhoea in children at PML but did not find any case of Giardiasis. We do not rule out the possibility that there could be other causes of diarrhoea in children [22, 34]. Assessment of clinical data on diarrhoea cases showed that over 70% of children presented with diarrhoea and vomiting, and more than 80% passed out loose watery stools. We suspect that there may be other underlying causes of these symptoms than *G. lamblia*. It would be interesting, in future studies, to determine if children presenting with diarrhoea may be co-infected with *Cryptosporidium spp* and *G. lamblia*. Also, we did not culture samples for presence of enteric pathogens, which have also been associated with diarrhoea [35].

Risk factors for giardiasis and other studies looking at the association between diarrhoea and some socio-demographic parameters, reviewed by Muhsen and Levine [36], have informed public health efforts in controlling infections and containing outbreaks. However, socio-economic, cultural and lifestyle differences imply that these findings may not necessarily be relevant to all social classes, particularly in developing countries where sanitation is a huge problem. In the current study, we wanted to understand how certain behavioural and socio-economic practices of mothers with children < 5 years, may pose as possible risk factors of *G. lamblia* infection. We observed that infection was independent of gender and age but seemed higher in ages 2-3 years (OR=3.456). These observations are parallel with two similar studies [37, 38]. Elsewhere, in Lagos, *G. lamblia* infection was common among the age group 4-5 years [39]. In any case, during outbreaks, infections within the studied population may spread faster, as it is during this age that children tend to play more outdoors and are likely to have frequent contact with each other. Public health education on good sanitary practices including frequent hand washing, particularly at antenatal and day-care centers, may contribute to controlling infection rates. In assessing the role of some socio-economic and other parameters on infection, we observed that the risk of *G. lamblia* infection was independent of, but may be higher in children whose mothers had little or no formal education, were fed with food sold on the streets and drunk sachet water. Two independent studies observed a similar trend on education of mothers and risk of infection in their children [40, 41], which suggest that well-educated mothers are likely to be better informed of good sanitary practices. Thus, they may also be aware of common food and water borne diseases and are more likely to prepare food at home for their children. After screening samples of different brands of sachet water, Kwakye-Nuako et al. [42] showed that > 70% of the samples were contaminated with microorganisms including oocysts of *Cryptosporidium* sp. Although several studies have reported the health benefits children derive from their mothers breast milk [36, 43], we did not see any protective role it had on children in this study. Our data agree with findings by Islam et al [44], who did not see a clear-cut protective role of human milk antibodies. These findings are interesting and warrant further investigations into the role of breast milk in giardiasis. Berrilli et al. [45] suggested that infection of humans by zoonotic genotypes from domestic animals could be of low epidemiological significance, although possible. In this study, > 50% of cases had domestic animals at home but we did not find any association between pet/animal ownership by mothers and *G. lamblia* infection in their children. However, the zoonotic potential of genotype B cannot be ruled out [46–48]; we observed that >5% of cases who had animals were infected with genotype B, though not statistically significant. We did not collect stool samples from animals to confirm possible presence of *G. lamblia*. On role of seasonality, we did not observe any significant difference between infections in rainy and dry seasons. Risk of infection may be influenced by both seasons as the cyst can survive temperature extremes [41, 49–52]. Improving general health conditions is key to controlling infections of Giardiasis but understanding common socio-cultural practices of a population may inform risk factors associated with the infection, considering the variation in behaviour and lifestyle.

## Conclusion

PCR and ELISA offered better detection of *G. lamblia* DNA in stool samples than microscopy. We recorded a lower prevalence of *G. lamblia* compared to previous studies, and our findings suggest that assemblage B could be the predominant genotype causing giardiasis in children.

### What is known about this topic

- The prevalence of Giardia is strongly associated with a variety of risk factors related to the host, such as socio-demographic, environmental and zoonotic conditions

### What this study adds

- Keeping of pets and other domestic animals in Ghanaian homes, which is a common practice, is unlikely to be a risk factor of G. lamblia infection;
- Assemblage B is probably the predominant genotype causing giardiasis in children in Accra
